# Bayesian bi-level variable selection for genome-wide survival study

**DOI:** 10.5808/gi.23047

**Published:** 2023-06-28

**Authors:** Eunjee Lee, Joseph G. Ibrahim, Hongtu Zhu, for the Alzheimer’s Disease Neuroimaging Initiative

**Affiliations:** 1 Department of Information and Statistics, Chungnam National University, Daejeon 34134, Korea; 2 Department of Biostatistics, University of North Carolina, Chapel Hill, NC 27599, USA

**Keywords:** Bayesian variable selection, genome-wide association studies, group structure, linkage disequilibrium, survival analysis

## Abstract

Mild cognitive impairment (MCI) is a clinical syndrome characterized by the onset and evolution of cognitive impairments, often considered a transitional stage to Alzheimer’s disease (AD). The genetic traits of MCI patients who experience a rapid progression to AD can enhance early diagnosis capabilities and facilitate drug discovery for AD. While a genome-wide association study (GWAS) is a standard tool for identifying single nucleotide polymorphisms (SNPs) related to a disease, it fails to detect SNPs with small effect sizes due to stringent control for multiple testing. Additionally, the method does not consider the group structures of SNPs, such as genes or linkage disequilibrium blocks, which can provide valuable insights into the genetic architecture. To address the limitations, we propose a Bayesian bi-level variable selection method that detects SNPs associated with time of conversion from MCI to AD. Our approach integrates group inclusion indicators into an accelerated failure time model to identify important SNP groups. Additionally, we employ data augmentation techniques to impute censored time values using a predictive posterior. We adapt Dirichlet-Laplace shrinkage priors to incorporate the group structure for SNP-level variable selection. In the simulation study, our method outperformed other competing methods regarding variable selection. The analysis of Alzheimer’s Disease Neuroimaging Initiative (ADNI) data revealed several genes directly or indirectly related to AD, whereas a classical GWAS did not identify any significant SNPs.

## Introduction

Mild cognitive impairment (MCI) is a clinical syndrome characterized by the onset and evolution of cognitive impairments. As 10%–15% of MCI patients develop Alzheimer's disease (AD) annually, MCI is commonly regarded as a transitional stage to AD. Identifying genetic characteristics among MCI patients who experience an accelerated progression to AD is important in enabling early diagnosis and facilitating drug discovery for AD. Genome-wide association studies (GWAS) are a standard tool for identifying single nucleotide polymorphisms (SNPs) associated with specific clinical conditions or outcomes. Researchers can delineate the genetic factors related to the rapid progression from MCI to AD as a phenotype in a GWAS by using the time of conversion from MCI to AD.

As approximately 500,000 to one million candidate SNPs exist, a GWAS deals with high-dimensional data, where the number of variables (SNPs in a GWAS) *p* is much greater than sample size *n*. A classical GWAS conducts several association tests, so called multiple testing, that examine an individual effect of each SNP on a clinical outcome. The classical GWAS has two major limitations. First, GWAS has a multiple testing issue, which requires adequate control of false positives. Typically, the significance level of each test is adjusted by a Bonferroni correction. The significance level widely accepted to determine “genome-wide significant” association is 5 × 10^–8^ [1,2], which is a strict threshold and makes genome-wide significance difficult to be achieved. Second, the GWAS does not account for the intricate group structure among SNPs such as genes or linkage disequilibrium (LD) blocks. LD reflects how much an allele from a particular genetic variant is associated or inherited with an allele from another nearby genetic variant within the same population [3]. Incorporating the group information within the GWAS would increase statistical power by aggregating small effects of SNPs within a group.

To resolve the multiple testing issue, many statistical methods have been developed in terms of penalization [4-7], Bayesian variable selection [8,9], and sure independence screening strategy [10-12]. Researchers proposed variable selection methods by incorporating the group information to select genetic variants in both gene and SNP levels simultaneously [13-16]. While these methods are suitable for a continuous or binary outcome, only a limited number of studies for a time-to-event outcome are available. Bi et al. [17] developed a saddlepoint approximation implementation to correct p-values based on the Cox regression model. A Bayesian survival model with variable selection was proposed with application to GWAS [18]. Lin et al. [19] proposed kernel-machine SNP-set analysis to assess the group effect of each SNP-set on the survival time.

We propose a Bayesian bi-level variable selection (BBVS) method to detect SNPs associated with a time-to-event outcome by considering all the SNPs simultaneously and incorporating the group information of the SNP data, based on an accelerated failure time (AFT) model. Our method has two hierarchical levels of variable selection: the first level is group-wise and the second level is element-wise variable selection. In the first level, we identify important groups of variables by employing group inclusion indicators in the AFT model and update the censored event time from its predictive posterior distribution by data augmentation [18,20,21]. As this step generates posterior samples of censored time to event, their posterior mean will be used as an imputed value for the censored event time in the second level. In the second level, we only include variables in the selected groups in the first level as covariates in the regression model. To conduct element-wise variable selection, we adapt Dirichlet-Laplace shrinkage priors [22] to incorporate the group structure.

The rest of this paper is organized as follows. In the “Methods” section, we discuss our BBVS in the AFT model. In the Results and Discussion section, we present simulation study results to validate and compare the performance of BBVS with other group/bi-level selection methods. We discuss the real data analysis results for Alzheimer’s Disease Neuroimaging Initiative (ADNI) data.

## Methods

### Accelerated failure time model

An AFT model is a parametric model to analyze a time-to-event outcome. While Cox regression postulates that covariates are multiplicatively related to the hazard, an AFT model assumes a direct relationship between time to event and covariates, which enables straightforward interpretation of regression coefficients. For the *i*-th subject,*Y_i_* is survival time and ***X**_i_* = (1,*x*_*i*1_,*x*_*i*2_, ⋯, *x_ip_*)' is a covariate vector. The first element of 1 allows estimation of the *y*-intercept. Subsequently, AFT model is given by $Y_{i}=\exp\left(x_{i}^{'}\beta \right)v_{i},\;i=1,\cdots ,\;n$. It becomes the linear model in a log scale $\log Y_{i}=x_{i}^{'}\beta +\epsilon_{i},\;i=1,\;\cdots ,n,$, where ***β*** = (*β*_0_,*β*_1_, ⋯, *β_p_*) is a vector of *p* + 1 unknown regression coefficients including the *y*-intercept of *β*_0_, and *ϵ_i_* = log *v_i_* is an error term. Generally, the error term is assumed to follow the parametric distribution, such as normal distribution. The parametric AFT model is discussed extensively in [23-26] of the frequentist framework. Bayesian approaches were developed for the parametric AFT model [27-29]. In this paper, we consider a parametric Bayesian approach to model the error term *ϵ_i_* with a normal distribution.

### BBVS in accelerated failure time model

We propose a BBVS method on the AFT model. This method has two hierarchical levels of variable selection, the group-wise and the element-wise variable selection. It is motivated by natural grouping structures of SNPs, which can be captured by genes or LD blocks. By making use of the group structure in the model frame, we can efficiently select a small number of SNPs associated with a time-to-event outcome.

With predefined *G* blocks we can write our model as follows.


(1) $$\log Y_{i}=x_{i,0}^{'}\beta_{0}+\sum_{g=1}^{G}\gamma_{g}x_{i,g}^{'}\beta_{g}+\epsilon_{i},i=1,\cdots ,n,\;$$

where $x_{i,0}=\left(1,x_{i,1}^{0},\cdots ,x_{i,p_{0}-1}^{0}\right),\beta_{0}=\left(\beta_{0,0},\beta_{0,1},\cdots ,\beta_{0,p_{0}-1}\right)$. For each *g*-th group of variables,$x_{i,g}=\left(x_{i,1}^{g},x_{i,2}^{g},\cdots ,x_{i,k_{g}}^{g}\right)$, $\beta_{g}=\left(\beta_{g,1},\beta_{g,2},\cdots ,\beta_{g,k_{g}}\right)$. Denote ***β*** = (*β*_1_, ⋯, *β_G_*), ***γ*** = (*γ*_1_, ⋯, *γ_G_*), where *γ_g_* is an indicator variable having 0 or 1. When *γ_g_* = 1, the *g*-th set of variables will be included in the model. If *γ_g_* = 0, we remove the *g*-th group in the model construction. The covariates $x_{i,1}^{0},\cdots ,x_{i,p_{0}-1}^{0}$ are included in the model to address their effects on the time to event. They can be clinical and demographic characteristics of subjects. The error term *ϵ_i_*’s are assumed to be independently distributed as *N*(0,*σ*^2^); hence, that the failure time *Y_i_* follows a log-Normal distribution. When *y_i_* is possibly right censored, we only observe *t_i_* = *min*(*Y_i_*,*c_i_*) and *ν_i_* = *I*{*y_i_*<*c_i_* }, where *c_i_* is the censoring time. Here *w_i_* = *log*(*y_i_*) can be considered as the augmented data such that


(2) $$\begin{array}{l} w_{i}=\;\log\left(t_{i}\right)\;\;if\;{\text{ν}}_{\text{i}}=1,\\ w_{i}>\;\log\left(t_{i}\right)\;\;if\;{\text{ν}}_{\text{i}}=0.\;\; \end{array}$$

Our bi-level variable selection method addresses two issues in the model (1): the selection of the relevant groups of SNPs and the imputation of the censored time to event *y_i_*. In the first step, we identify important groups of variables by updating only the group inclusion vector *γ* and the censored time *y_i_* from their posterior distributions. In the second step, the model (1) can be reduced by


(3) $$\log Y_{i}=x_{i,0}^{'}\beta_{0}+\sum_{g=1}^{Q}\gamma_{g}x_{i,g}^{*}{}^{'}\theta_{g}+\epsilon_{i},i=1,\cdots ,n,$$

where $x_{i,g}^{*},\;\;g=1,2,\cdots ,Q$ are the *Q* selected groups in the first step, and ***θ**_g_*, *g* = 1, 2, ⋯, *Q* are the corresponding regression coefficient vectors. The censored time to event *y_i_* is imputed by the mean of the posterior samples of *w_i_* collected in the first step. It converts the AFT model to a usual log-linear regression problem.

We employ a shrinkage prior on the regression parameters ***θ**_g_* to enable the element-wise variable selection within $x_{i,g}^{*}{}^{'},g=1,2,\cdots ,Q$. We consider a Dirichlet-Laplace (DL) prior proposed by Bhattacharya et al. [22] on the regression parameters and extend it to incorporate grouping information. As the regression parameters ***β***_0_,***β*** and the standard deviation *σ* of the error term are not of interests, the computational burden in the first step can be reduced by integrating out the irrelevant parameters, ***β***_0_, ***β***, *σ* from the full posterior distribution. This kind of strategy has been employed in Sha et al. [20], although their variable selection has been conducted only in an element-wise fashion.

#### The first step: group-wise variable selection

In the first step, we consider the following conjugate priors.


$$\begin{array}{l} \left.\beta_{0}\right|\sigma^{2}\;\;\;\sim \;\;\;N\left(0,\;\sigma^{2}h_{0}I_{p_{0}}\right)\\ \left.\beta_{g}\right|\sigma^{2}\;\;\;\sim \;\;\;N\left(0,\;c_{0}\sigma^{2}\;\sum_{g}\right),\;g=1,\cdots ,\;G\\ \sigma^{2}\;\;\;\sim \;\;\;IG\left(v_{0}/2,\;v_{0}\sigma^{2}{}_{0}/2\right)\\ \gamma_{j}\;\;\;\sim \;\;\;Bernoulli\left(p_{j}\right)\\ p_{j}\;\;\;\sim \;\;\;Beta\left(a,\;b\right) \end{array}$$

In the prior, $x_{0}={\left[x_{1,0},\cdots ,x_{n,0}\right]}^{'}$, $x_{g}={\left[x_{1,g},\cdots ,x_{n,g}\right]}^{'}$, $X=\left[X_{0},X_{1},\cdots ,X_{G}\right]$, $\Sigma_{g}={\left(x_{g}^{'}x_{g}\right)}^{-1}$ when *k_g_*≤*n* and $\Sigma_{g}={\left(x_{g}^{'}x_{g}+\lambda I_{k_{g}}\right)}^{-1}$ when *k_g_* > *n* for the *g*-th group with size *k_g_*. The prior on ***β**_g_* is the Information Matrix (IM) or Information Matrix Ridge prior proposed by Gupta and Ibrahim [30]. It is a generalization of Zellner's g-prior [31], while the IM prior is equal to the Zellner's g-prior in the Gaussian linear regression setting. The full posterior distribution of (***β**_g_*, ***β***, *γ*, *σ*^2^) is given by


$$L(\beta_{0},\;\beta ,\;\gamma ,\;\sigma^{2}|w,\;x)\;\propto \;L(w|x,\;\beta_{0},\;\beta ,\;\sigma^{2},\;\gamma )\pi (\beta_{0}|\sigma^{2})\pi (\beta |\sigma^{2})\pi (\gamma )\pi (\sigma^{2})\;\propto \;(\sigma^{2}{)}^{-n/2}\exp\{-\frac{1}{2\sigma^{2}}\sum_{i=1}^{n}(w_{i}-x_{i0}^{'}\beta_{0}-\sum_{g=1}^{G}\gamma_{g}x_{i,g}^{'}\beta_{g}{)}^{2}\}\times {{(\sigma }^{2})}^{-p_{0}/2}exp\{-\frac{1}{2h_{0}\sigma^{2}}\beta_{0}^{'}\beta_{0}\}\;\times \prod_{g=1}^{G}{{(\sigma }^{2})}^{-k_{g}/2}\exp\{-\frac{1}{2c_{0}\sigma^{2}}\beta_{g}^{'}\sum_{g}^{-1}\beta_{g}\}\times (\sigma^{2}{)}^{-\nu_{0}/2-1}\exp(-\frac{\nu_{0}\sigma_{0}^{2}}{2\sigma^{2}})\;\times \prod_{g=1}^{G}p_{g}^{\gamma_{g}}(1-p_{g}{)}^{1-\gamma_{g}}\times \prod_{g=1}^{G}\frac{1}{B(a,b)}p_{g}^{a-1}(1-p_{g}{)}^{b-1}$$

By integrating out ***β***_0_, ***β***, *σ*^2^, we can obtain the posterior distribution of ***γ*** :


$$L\left(\gamma \text{|}w,\;x\right)\propto {\left\{\nu_{0}\sigma_{0}^{2}+w^{\prime }{\left(I+h_{0}x_{0}x_{0}^{'}+c_{0}\overset{G}{\underset{g=1}{\sum }}\gamma_{g}x_{g}\sum_{g}x_{g}^{'}\right)}^{-1}w\right\}}^{-\frac{n+\nu_{0}}{2}}\times \prod_{g=1}^{G}p_{g}^{\gamma_{g}}{\left(1-p_{g}\right)}^{1-\gamma_{g}}$$

For a given ***γ***(g) = (*γ*_1_, ⋯, *γ*_*g*-1_,*γ*_*g*+1_, ⋯, *γ_G_*), the posterior distribution of *γ_g_* is the Bernoulli distribution with success probability $\frac{A}{A+B}$, where


$$\begin{array}{l} A=\;f_{t}(w|\nu_{0},\;\sigma_{0}\left(A_{\gamma_{\left(g\right)}}+c_{0}\gamma_{g}x_{g}\sum_{g}x_{g}^{'}\right))\times p_{g},\\ B=\;f_{t}(w|\nu_{0},\;\sigma_{0}A_{\gamma_{\left(g\right)}})\times (1-p_{g}), \end{array}$$

and $A_{\gamma_{\left(g\right)}}=I+h_{0}x_{0}X_{0}^{'}+c_{0}\sum_{k\neq g}^{G}\gamma_{k}X_{k}\Sigma_{k}X_{k}^{'}$. The function *f_t_* (∙|*ν*,*σ*^2^) denotes the probability density function of t-distribution with the degrees of freedom *ν* and the scale parameter *σ*^2^. Then, update p_g_ from its posterior distribution Beta (*a* + *γ_g_*, *b* + 1 - *γ_g_*). The marginal likelihood of the augmented data *w* can be derived as


$$L\left(w\text{|}X,\gamma \right)\propto {\left\{1+\frac{1}{\nu_{0}\sigma_{0}^{2}}w^{\prime }{\left(I+h_{0}X_{0}X_{0}^{'}+c_{0}\overset{G}{\underset{g=1}{\sum }}\gamma_{g}X_{g}\sum_{g}X_{g}^{'}\right)}^{-1}w\right\}}^{-\frac{\nu_{0}+n}{2}},$$

which is proportional to the truncated *n*-dimensional multivariate t-distribution with truncation given by (2) as follows.


$$w|Χ,\gamma \sim t_{n}\left[\nu_{0},\;0,\sigma_{0}^{2}\left(I+h_{0}X_{0}X_{0}^{'}+c_{0}\overset{G}{\underset{g=1}{\sum }}\gamma_{g}{Χ}_{g}\sum_{g}X_{g}^{'}\right)\right]$$

By using the full conditional distribution of *w_i_* for a censored case *ν_i_* = 0, the censored time *w_i_* can be imputed by its posterior mean. Denote $H_{\gamma }=I+h_{0}X_{0}X_{0}^{'}+c_{0}\sum_{g=1}^{G}\gamma_{g}{Χ}_{g}\sum_{g}X_{g}^{'}$, where *h_i,j_* is a scalar element in *i*-th row,*j*-th column of *H_γ_* and *H*_(*i,j*)_ is the matrix *H_γ_* without its *i*-th row and *j*-th column, and $h_{i}^{\left(i\right)}$ is the *i*-th row of *H_γ_* without its *i*-th element. Similarly, let ***w***^(*i*)^ be the vector ***w*** without its *i*-th element. When *w_i_* is censored, its full conditional distribution can be written as a truncated *t* location-scale distribution such that


(4) $$w_{i}|w^{\left(i\right)},X,\gamma ∼t_{n+\nu_{0}-1}\left(\mu_{w_{i}},\;s_{w_{i}}\right),\;w_{i}\rangle \log\left(t_{i}\right)$$

where *μ_wi_*, *s_wi_*, and *n*+*ν*_0_-1 are respectively the location, scale, and degrees of freedom parameters. The location and scale parameters are given by


$$\begin{array}{l} \mu_{w_{i}}=\;h_{i}^{\left(i\right)}{Η}_{\left(i,j\right)}^{-1}w_{i}^{\left(i\right)},\\ s_{w_{i}}\;=\;\sqrt{\left(h_{\left(i,i\right)}-h_{i}^{\left(i\right)}{Η}_{\left(i,i\right)}^{-1}h_{i}^{{\left(i\right)}^{'}}\right)\left(\nu_{0}\sigma_{0}^{2}+w_{i}^{\left(i\right)}{Η}_{\left(i,i\right)}^{-1}w_{i}^{\left(i\right)}\right)/\left(n+\nu_{0}-1\right).} \end{array}$$

The censored *w_i_* will be updated from (4) at each iteration and it will be imputed as their posterior mean in the element-wise selection step.

After running Gibbs sampling with *M* iterations, posterior inclusion probability can be calculated from the posterior sample of *γ* as their posterior mean, $\hat{p_{g}}=\frac{1}{M}\sum_{m=1}^{M}\gamma_{g}^{\left(m\right)}$. The posterior inclusion probability $1-\hat{p_{g}}$ can be considered as Bayesian q-values, or estimates of the local false discovery rate (FDR) [32,33], because they measure the probability of a false positive if the *g*-th group is "decided" to be included in the model. To select important groups, for some threshold p^*^, we consider that any group with $\hat{p_{g}}\geq p^{*}$ is relevant and will include them in our model. We determined the threshold p^*^ to control the average Bayesian FDR by using the method proposed by Morris et al. [34].

#### The second step: element-wise variable selection

In this step, we include all the variables of the *Q* selected groups in the first step and assume shrinkage priors on the regression parameters ***θ***_1_, ⋯, ***θ**_Q_* to achieve further sparsity in the element-wise level in the reduced model (3). As a shrinkage prior, the DL prior is assumed and extended to incorporate grouping information. The DL prior has been proposed in [22] as a novel form of shrinkage prior. Under the normal means setting


$$y_{i}=\theta_{i}+\epsilon_{i},\epsilon_{i}∼N\left(0,1\right),\;1\leq i\leq p,$$

the true signal *θ_i_* has a DL prior, which has a hierarchical structure such that


(5) $$\theta_{j}|\psi_{j},\phi_{j},\tau \sim N\left(\psi_{j}\phi_{j}{}^{2}\tau^{2}\right),\;\;\psi_{j}\sim Exp\left(1/2\right),\phi \sim Dir\left(a,\cdots ,a\right),\;\tau \sim Gamma\left(pa,\;1/2\right),$$

where ***ϕ*** = (*ϕ*_1_, *ϕ*_2_, ⋯, *ϕ_p_*). To efficiently control the global shrinkage, they introduced global (*τ*) and local (***ϕ***) scales, where the local scales have a joint structure such that they lie in the (*p*-1) dimensional simplex. Under the moderate-sized coefficients with sparse signal setting, their simulation study has shown that the DL prior outperforms least absolute shrinkage and selection operator (Lasso), Bayesian Lasso, empirical Bayes median, and point mass prior, while its performance is similar to that of Horseshoe prior.

In our model framework, we have prespecified grouping information. In order to get more flexibility depending on the grouping structure, we allow the hyperparameters *ψ_j_*, *ϕ_j_*, and *τ* in (5) to be group index(*g*)-dependent. In the selected group *g*, there are *q_g_* variables and the total number of selected variables in the model (3) is $q=\sum_{g=1}^{Q}q_{g}$. Here, we impute *w* by the posterior mean $\tilde{w}$ obtained from the group-wise selection step. For *g* = 1, 2, ⋯, *Q*, the priors are set to be


(6) $$\begin{array}{l} \theta_{g}|\sigma^{2},\psi_{g},\phi_{g},\tau_{g}\;\sim \;\;N\left(0,\sigma^{2}\Sigma_{g}^{*}\right)\\ \sigma^{2}\;\sim \;\;IG\left(\nu_{0}/2,\nu_{0}\sigma_{0}^{2}/2\right)\\ \psi_{gj}\;\sim \;Exp\left(1/2\right),\;j=1,\cdots ,q_{g}\\ \left(\phi_{g1},\cdots ,\phi_{gq_{g}}\right)\;\sim \;\;Dir\left(a_{g},\cdots ,a_{g}\right)\\ \tau_{g}\;\sim \;\;gamma\left(q_{g}a_{g},1/2\right)\\ a_{g}\;\sim \;\text{Discrete uniform from}\;\frac{1}{q_{g}}\;\text{to}\frac{1}{2}\text{with length}\;50, \end{array}$$

where $\Sigma_{g}^{*}=diag\left(\psi_{g1}\phi_{g1}^{2}\tau_{g}^{2},\cdots ,\psi_{gq_{g}}\phi_{gq_{g}}^{2}\tau_{g}^{2}\right),\psi_{g}=(\psi_{g1},\cdots ,\;\psi_{gq_{g}}),\;\phi_{g}=(\phi_{g1},\cdots ,\;\phi_{gq_{g}})$. Here *IG*(*α,b*) denotes the inverse gamma distribution with shape parameter *α* and the rate parameter *b*.

Denote xi*'=x*i,1', ⋯, x*i,Q',θ'=θ1',⋯,θQ',  ϕ'=ϕ′1,⋯,ϕ′Q,  ψ'=ψ'1,⋯,ψ'Q, and ***τ*** = (*τ*_1_, ⋯, *τ_Q_*). The design matrix is given by $X_{g}^{*}={\left[x_{1,g}^{*},,\cdots ,x_{n,g}^{*}\right]}^{'}$ for each *g*, and $X^{*}=\left[X_{1}^{*},\cdots ,X_{Q}^{*}\right]$ for all the groups. Σ^*^ is a block diagonal matrix with element matrices $\Sigma_{1}^{*},\;\cdots ,\;\Sigma_{Q}^{*}$. By combining (3) and (6), the posterior distribution can be obtained as


(7) $$L(\beta_{0},\;\theta ,\;\sigma^{2},\;\phi ,\;\psi ,\;\tau |\tilde{w},\;X^{*})\;\propto \;L(\tilde{w}|X_{*},\;\beta_{0},\;\theta ,\;\sigma^{2})\pi (\beta_{0}|\sigma^{2})\pi (\theta |\sigma^{2},\;\phi ,\;\psi ,\;\tau )\pi (\sigma^{2})\pi (\phi )\pi (\psi )\pi (\tau )\;\propto \;(\sigma^{2}{)}^{-n/2}\exp\{-\frac{1}{2\sigma^{2}}\sum_{i=1}^{n}(\tilde{w_{i}}-x_{i0}^{'}\beta_{0}-x_{i*}^{'}\theta {)}^{2}\}\times exp\{-\frac{1}{2h_{0}\sigma^{2}}\beta_{0}^{'}\beta_{0}\}\;\times \;\det(\sigma^{2}\sum^{*}{)}^{-1/2}\exp\{-\frac{1}{2\sigma^{2}}\theta^{'}\sum^{*-1}\theta \}\times (\sigma^{2}{)}^{-\nu_{0}/2-1}\exp(-\frac{\nu_{0}\sigma_{0}^{2}}{2\sigma^{2}})\;\times \;exp(-\frac{\sum_{g=1}^{Q}\sum_{j=1}^{q_{g}}\psi_{gj}}{2})\times \prod_{g=1}^{Q}(\frac{1}{B(\phi_{g})}\prod_{j=1}^{q_{g}}\phi_{gj}^{a_{g}-1})\times \prod_{g=1}^{Q}\{\tau_{g}^{q_{g}a_{g-1}}\exp(-\frac{\tau_{g}}{2})\},$$

where *B*(*ϕ_g_*) denotes a multivariate Beta function. We propose a Gibbs sampler for posterior computation, which enables parameter estimation and variable selection simultaneously. The Gibbs sampler is computationally efficient and mixes rapidly. We first specified the hyperparameters *h*_0_, *σ*_0_, *ν*_0_, *a*_1_, ⋯, *a_g_* at appropriate values. Starting from the initiation step, the Gibbs sampler for the model (3) and (7) proceeds as follows:

1. Update *β*_0_ according to its full conditional distribution


$$p\left(\beta_{0}\text{|}-\right)∼N_{p_{0}}\left({\left(X_{0}^{'}X_{0}+\frac{1}{h_{0}}I\right)}^{-1}X_{0}^{'}\left(\tilde{w}-X^{*}\theta \right),\sigma^{2}{\left(X_{0}^{'}X_{0}+\frac{1}{h_{0}}I\right)}^{-1}\right).$$

2. Update *θ_g_* from its full conditional distribution $N_{q_{g}}\left(\tilde{\mu_{g}},{\tilde{\Sigma }}_{g}\right)$, where


$$\begin{array}{l} \tilde{\mu_{g}}=\;{\left(X_{g}^{*'}X_{g}{}^{*}+\Sigma_{g}^{*}\right)}^{-1}X_{g}^{*'}\left(\tilde{w}-X_{0}\beta_{0}-X_{\left(g\right)}^{*}\theta_{\left(g\right)}\right),\\ {\tilde{\Sigma }}_{g}=\;\sigma^{2}{\left(X_{g}^{*'}X_{g}^{*}+\Sigma_{g}^{*}{}^{-1}\right)}^{-1}. \end{array}$$

The design matrix $X_{\left(g\right)}^{*}$ is $\left[X_{1}^{*},\cdots ,X_{g-1}^{*},X_{g+1}^{*},\cdots ,X_{Q}^{*}\right]$, and the regression coefficient vector $\theta_{\left(g\right)}^{'}$ is ($\left(\theta_{1}^{'},\cdots ,\theta_{g-1}^{'},\theta_{g+1}^{'}\cdots ,\theta_{Q}^{'}\right)$).

3. Let *N* = *n*+*q*+*p*_0_+*ν*_0_ and $\eta =X_{0}^{}\beta_{0}+X^{*}\theta$. Update *σ*^2^ from


$$p\left(\sigma^{2}\text{|}-\right)∼IG\left(\frac{N}{2},\;\frac{1}{2}\left\{\nu_{0}\sigma_{0}^{2}+\tilde{w}-\eta^{2}+\frac{\beta_{0}^{'}\beta_{0}}{h_{0}}+{\left(\Sigma^{*}\right)}^{-1}\theta \right\}\right)$$

4. Independently sample *ψ_gj_* from its full conditional distribution


$$p\left(\psi_{gj}\text{|}-\right)∼IG\left(\frac{\phi_{gj}\tau_{g}\sigma }{\left|\theta_{gj}\right|},1\right).$$

5. Update *τ_g_* from its full conditional distribution, the generalized inverse Gaussian distribution (giG), such as


$$p\left(\tau_{g}\text{|}-\right)∼\text{giG}\left(q_{g}\times a_{g}-q_{g},1,2\sum_{j=1}^{q_{g}}\frac{\left|\theta_{gj}\right|}{\phi_{gj}\sigma }\right).$$

6. Update *ϕ_gj_*, where *ϕ_gj_* = *T_gj_*/*T_g_* such that


$$p\left(T_{gj}\text{|}-\right)∼\text{giG}\left(a_{g}-1,1,2\frac{\left|\theta_{gj}\right|}{\sigma }\right).$$

7. Update *a_g_* from MN($1,\tilde{p_{1}}/\tilde{p},\cdots ,\tilde{p_{50}}/\tilde{p}$), where $\;\tilde{p}=\sum_{l=1}^{50}\tilde{p_{l}}$ and


$$\tilde{p}=\exp\left(\left(u_{l}-1\right)\sum_{j=1}^{q_{g}}log\left(\phi_{gj}\right)+\left(q_{g}u_{l}-1\right)\log\left(\tau_{g}\right)-\log50\right).$$

As the DL prior does not give exactly zero coefficient value, an additional step is needed to select relevant variables. We followed a simple approach to choose important variables using k-means clustering [22]. Two clusters of |*θ_j_* |'s can exist, where (a) one cluster has nearly zero coefficient values while (b) another cluster has relatively bigger absolute coefficients away from zero. The clusters (a) and (b) can be considered as noise and signal, respectively. We cluster |*θ_j_* |'s at each Markov chain Monte Carlo iteration using k-means with *k* = 2 clusters. At each *i*-th iteration, the number of important variables *h_i_* is set to be the smaller cluster size out of the two clusters. Subsequently, the number of important variables is finally estimated by taking the mode from the whole Markov chain Monte Carlo (MCMC) iterations, i.e.,*H* = mode{*h_i_*}. The *H* largest elements of the absolute values of posterior medians |*θ*| are identified as the important variables.

### ADNI-1 data

To reveal SNPs associated with the time of conversion to AD from MCI, we analyzed ADNI data obtained from the ADNI database (https://adni.loni.usc.edu). The ADNI was launched in 2003 as a public-private partnership, led by Principal Investigator Michael W. Weiner, MD. The primary goal of ADNI has been to test whether serial magnetic resonance imaging, positron emission tomography, other biological markers, and clinical and neuropsychological assessment can be combined to measure the progression of MCI and early AD. The initial 5-year ADNI study resulted in the ADNI-1 data.

We performed quality control (QC) steps on the raw genotype data to ensure that only high-quality data were included in the final analysis. QC procedures include (1) call rate check per subject and per SNP marker, (2) gender check, (3) sibling pair identification, (4) the Hardy-Weinberg equilibrium test, (5) marker removal by the minor allele frequency, and (6) population stratification. The second line preprocessing steps include removal of SNPs with (1) more than 5% missing values, (2) minor allele frequency smaller than 5%, and (3) Hardy-Weinberg equilibrium p-value <10^-6^. The remaining missing genotype variables were imputed as the modal value. After the QC procedures, 347 subjects and 494,564 SNPs remained in the current study. The above procedures were carried out in PLINK version 1.9. We also calculated the LD blocks to form the SNP-sets and remove SNP-sets with a single SNP. Eventually, 421,823 SNPs were left in our analysis grouped into 16,084 SNP-sets.

We study the subjects diagnosed with MCI at the baseline visit. If an MCI patient does not progress to AD within 48 months from the baseline, we define the time of conversion of the patient as "censored." For non-censored cases, the conversion time is determined by the difference between the baseline and the time of visit when the patient was diagnosed with AD.

### Simulation data

We generated simulation data to examine the performance of the BBVS in the AFT model. To convey the correlation structure of SNP data in practice, our SNP data is simulated from the Hapmap projects 2009 phase III data [35]. For each subject, we randomly combined two haplotypes from the Centre d'étude du polymorphisme humain population to form its genotypes and used PLINK [36] to form SNP-sets by determining LD blocks. Among the blocks that were >30, we randomly selected 2,000 SNP-sets in each block, which results in about 86,000 SNPs. After removing those SNP data with duplicated columns, we have about 45,000 SNPs in total.

We considered two cases: non-censored data and censored data. In the non-censored case, the time to event outcome was generated from the model (1), where $\gamma_{j}=1,\;\;j=1,\cdots ,10\;$ and $\gamma_{j}^{'}=0,\;j^{'}=11,\cdots ,2,000$. Within the 10 relevant blocks, we randomly selected 10 SNPs and assumed an additive model. The additive model assumes that a uniform, linear increase in risk for each copy of the minor allele exists. The corresponding non-zero regression coefficients were generated from *N*(-1, 0.5), which mimics the situation wherein a single copy of the minor allele decreases the time to event in relation to major allele. In the censored case, the censored event times were independently generated from a uniform distribution from 0 to c^*^. The value of c^*^ was set to achieve a desired censoring rate. We replicated the simulation 50 times under the same setting. We assumed the inclusion indicator *γ_g_*∼"*Beta*" (10,190), which gives average 5% of inclusion probability to reflect prior information that the important signal is sparse in the GWAS.

## Results and Discussion

### ADNI-1 data analysis

We applied BBVS on the ADNI-1 data to reveal SNPs associated with the time of conversion to AD from MCI. Other than the whole SNPs data, we also included demographic and clinical characteristics measured at the baseline, such as gender, age, handedness, marital status, education length, retirement, and Alzheimer’s Disease Assessment Scale–Cognitive Subscale (ADAS-Cog) score. The first five principal components would adjust for population stratification in the model [37]. The variable selection was only performed on the SNP data.

**Fig. 2.**
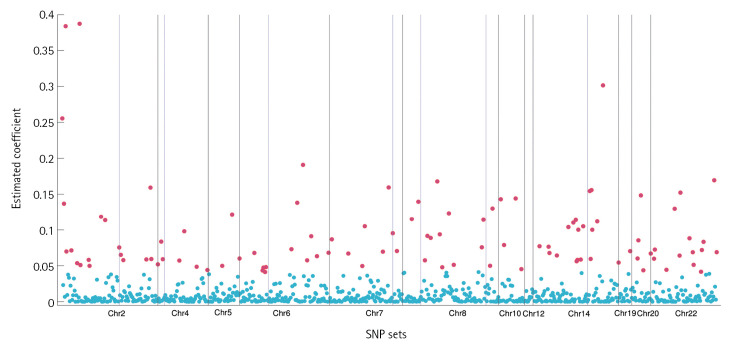
Estimated coefficient values for 795 SNPs included in the 19 SNP-sets. We colored 106 SNPs selected in the element level in red. SNP, single nucleotide polymorphism.

We determined the threshold *α* to control the average Bayesian FDR [34] and consider any group whose posterior inclusion probability is greater than that of α. In the ADNI data, the threshold is calculated by 0.941 (cFig. 1). In total, 19 SNP-sets were detected as important groups and 106 SNPs were identified by the elementwise-level selection. Fig. 2. shows the estimated coefficient values for 795 SNPs included in the 19 SNP-sets. We colored 106 SNPs selected in the element level in red.

Supplementary Figs. 1–3 show trace plots of the regression coefficients *θ*_1,1_, *θ*_1,2_, and *θ*_1,3_ of the first selected SNP-set for 5,000 iterations of the MCMC algorithm. They show fast convergence of the algorithm, indicating its good mixing properties.

We summarized the variable selection results of BBVS to present which genes are involved in Table 1. Among them, four genes have been reported in other studies to be related to AD directly or indirectly. Dipeptidyl-peptidase 10 (DPP10) is known to modulate the electrophysiological properties, cell-surface expression, and subcellular localization of voltage-gated potassium channels [38]. Chen et al. [39] demonstrated that aggregation of DPP10 was related to neurodegenerative disorders including AD, diffuse Lewy body disease, and fronto-temporal dementia. In addition, DPP10 had robust reactivity within neurofibrillary tangles and plaque-associated dystrophic neurites in AD brains, which suggested that it is involved in the pathology of AD [40]. All the findings indicate that DPP10 is associated with a risk to develop AD in a direct or indirect manner. THSD7B has been reported to be associated with age-related cognitive decline based on repeated measures of 17 cognitive tests [41]. In addition, several linkage mappings have identified VPS26A to be associated with AD [42]. Sidekick cell adhesion molecule 1 was reported as a susceptibility gene for hypertension in Japanese individuals [43], where hypertension moderately increased risk of AD [44].

**Fig. 3.**
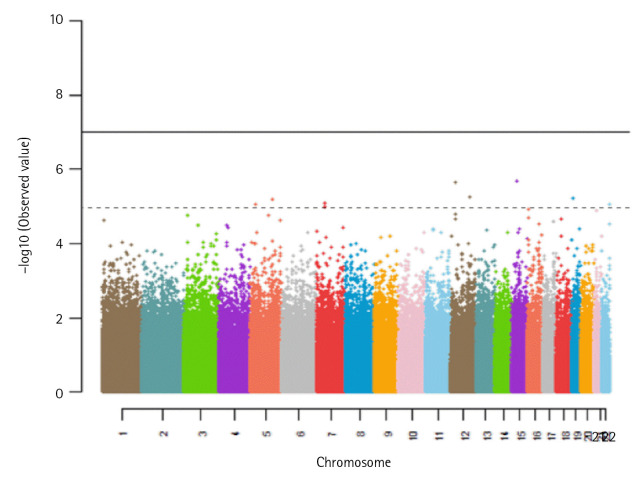
A Manhattan plot with –log10(p-value) for the classical genome-wide association study. The solid and dotted lines show the 5 × 10^-8^ significance level and the 1 × 10^-5^ significance level, respectively.

For comparison purposes, we conducted two different types of GWASs: (1) a simple GWAS, multiple testing on each SNP and (2) kernel-machine SNP-set GWAS with the linear kernel [19]. Fig. 3. shows a Manhattan plot with –log 10(p-value) for the simple GWAS. The solid and dotted lines represent the genome-wide significance level and the suggestive significance level, respectively. Our study identified 9 SNPs at the 1 × 10^-5^ suggestive significance level, where none of them had been reported in previous GWASs. Supplementary Table 1 shows the p-values of 106 SNP selected by the element-wise variable selection of the proposed method. None of them were significant at the suggestive significance level. For the kernel-machine method, we considered three types of kernel functions such as the linear kernel, the identical by state (IBS) kernel, and the quadratic kernel. Table 2 shows the SNP-sets selected by the kernel-machine method at the 5 × 10^-8^ significance level. The selected SNP-sets vary with the type of kernel. The linear kernel, the IBS kernel, and the quadratic kernel selected 5, 8, and 6 SNP-sets, respectively. Two genes, calmodulin-binding transcription activator 1 (*CAMTA1*) and *RBFOX1*, were related to Alzheimer’s disease. The linear kernel selected an SNP set located within *CAMTA1*. Huentelman et al. [45] identified SNPs within the *CAMTA1* gene that were significantly related to memory performance and memory-related regions on the human brain, which could be considered potential biomarkers of AD. *RBFOX1* was identified under the quadratic kernel function. Hooli et al. [46] reported that the gene co-segregates with disease status within early-onset familial AD and early or mixed-onset AD families. There were no overlapped SNPs among the three methods.

### Simulation study

As the AFT model for non-censored data is the log-normal regression model, we can compare the performance of variable selection with other variable selection methods implemented based on the typical regression models. For competing methods, we considered the group Lasso (grLasso) [47], the group MCP(grMCP) [48], the group bridge (gBridge) [49], the group exponential lasso (gel) [50], the composite MCP (cMCP) [51] penaltues. The cMCP, gel, and gBridge penalties carry out bi-level selection, meaning that they carry out variable selection at the group level and at the level of individual covariates. The grLasso, grMCP, and grSCAD penalties carry out variable selection only at the group level, meaning that within a group, coefficients will either all be zero or all non-zero. We used Bayesian Information Criteria to select the tuning parameter value for each method.

We consider the following performance measurements: true positive rate (*TPR* or sensitivity), true negative rate (*TNR* or specificity), positive predictive value (*PPV*), and negative predictive value (*NPV*). They are defined as follows.


$$TPR=\frac{TP}{10},TNR=\frac{TN}{1990},PPV=\frac{TP}{TP+FP},NPV=\frac{TN}{TN+FN},$$

where the *TP* and *TN* are the number of correctly identified significant variables and the number of correctly rejected non-significant variables, respectively. The *FP* and *FN* denote the number of identified non-significant variables and the number of rejected significant variables, respectively. Under the true model, *TP* = 10, *TN* = 1990, and *FP* = *FN* = 0, which implies that all the four rates are equal to one.

Table 3 shows the group-level and element-level variable selection results for the non-censored case. The average values of the performance measurements are presented with Monte Carlo standard errors in the parenthesis. Our method achieves the highest values of all the criteria,*TPR*, *TNR*, *NPV*, and *PPV* compared with other group penalty methods by removing the irrelevant groups consistently and selecting important groups very well. As the group penalties with only group-level selection especially grSCAD, grLasso tend to select groups more generously, they select important groups perfectly while the numbers of true positive cases are much bigger than other methods. The bi-level selection penalties, gBridge, and gel show comparative performance to our proposed method. In terms of the element-wise variable selection, our method yields the highest values of all the criteria,*TPR*, *TNR*, *NPV*, and *PPV* compared with other group penalty methods enabling bi-level selection. As the important signals are sparse, all the bi-level methods perform very well in terms of removing irrelevant signals.

The BBVS also shows satisfactory performance in terms of selecting important variables in the censored case. The average values of *TPR, TNR, PPV*, and *NPV* for the group-level selection from the 50 repetition of the simulation are 0.970, 1.000, 1.000, and 1. The corresponding Monte Carlo standard errors are 0.009, 0.000, 0.000, and 0.000. The average values of *TPR, TNR, PPV*, and *NPV* for the element-level selection are 0.634, 0.999, 0.589, and 1.000. The corresponding Monte Carlo standard errors are 0.012, 0.000, 0.011, and 0.000. Compared with non-censored cases, the performance of BBVS is satisfactory in the censored case as well.

### Conclusion

The BBVS was developed to enable bi-level variable selection as incorporating grouping information within covariates in the high-dimensional setting. In the context of GWAS, our method addressed the challenging issues by making use of natural grouping information of SNPs in the group-level variable selection step. In addition, DL priors were adapted to reflect the grouping information in the element-wise variable selection.

The simulation studies showed that our proposed method outperformed other bi-level and group-level variable selection methods in the GWAS setting for a non-censored case. We applied BBVS on the ADNI-1 data to identify relevant SNP-sets associated with the time to develop AD within MCI patients. We identified 106 informative SNPs located within 10 genes, where four genes were directly and indirectly related to AD, while the simple form of GWAS only detected 3 SNPs that had not been reported in the literature. We need to analyze other AD data sets to see if the implicated genes are reproducible when we used different subjects in the future study. We also need to conduct a simulation study to compare the variable selection performance of BBVS with other survival models that enable variable selection for the high-dimensional data.

## Figures and Tables

**Fig. 1.**
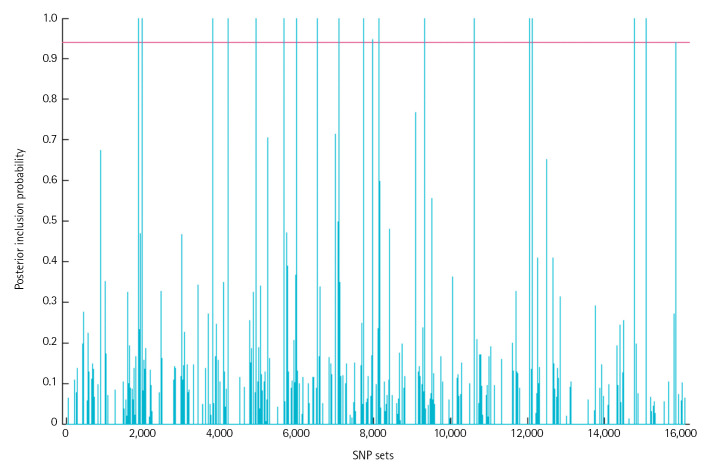
Posterior inclusion probabilities of 16,106 SNP-sets. Our proposed method identified 19 important SNP-sets after Bayesian FDR correction. The solid line shows the FDR criteria, 0.941 in this data. SNP, single nucleotide polymorphism; FDR, false discovery rate.

**Table 1.** LD blocks and genes detected by BBVS

Chr	Begin (bp)	End (bp)	No. of SNPs	No. of selected	Genes
2	50596	50665	70	13	*DPP10*
2	53530	53576	47	6	*THSD7B*
4	104778	104785	8	2	*ATP8A1*
4	117728	117780	53	4	*FREM3, LOC101927636, GYPA*
5	135218	135255	38	3	-
6	154825	154859	35	5	-
6	166701	166774	74	7	-
7	181879	181955	77	7	*SDK1*
7	197664	197675	12	1	-
8	216741	216762	22	2	*PREX2*
8	224172	224250	79	11	*TNFRSF11B, COLEC10*
8	228413	228427	15	2	*TRAPPC9*
10	261007	261038	32	4	*SRGN, VPS26A, SUPV3L1, HKDC1*
12	294754	294763	10	0	*CLEC2A, KLRF2*
14	332351	332416	66	12	*HEATR5A, DTD2, NUBPL*
14	334919	334956	38	7	-
19	394149	394164	16	1	*CPAMD8, HAUS8, MYO9B*
20	399491	399513	23	5	-
22	22468984	22671741	80	14	*VPREB1, BMS1P20*

LD, linkage disequilibrium; BBVS, Bayesian bi-level variable selection; SNP, single nucleotide polymorphism.

**Table 2.** LD blocks and the corresponding SNPs detected by the kernel-machine method

Chr	SNP	Gene	p-value		
			Linear	IBS	Quadratic
1	rs12128469, rs12402763, rs7543711, rs12563394, rs2301461, rs2301462	*CAMTA1*	5.00e-09	1.00e-04	6.00e-04
2	rs6545731, rs10169309		5.00e-09	2.00e-04	5.00e-09
2	rs2576778, rs880427	*FHL2*	4.00e-04	5.00e-09	2.10e-03
3	rs9288812, rs10511245, rs2053627		5.00e-09	3.00e-04	6.00e-04
3	rs6796883, rs293779	*CPNE9*	5.00e-04	5.00e-09	5.00e-03
3	rs307560, rs307558	*SYN2*	2.00e-04	5.00e-09	1.10e-03
3	rs6768031, rs1033222, rs1991443, rs1991442, rs1427840, rs11922896, rs6439279, rs748155, rs17275526, rs755568, rs1863916	*NEK11*	2.00e-04	5.00e-09	1.10e-03
5	rs6888634, rs2577531		5.00e-09	5.00e-09	2.00e-04
9	rs6475646, rs10733377		2.00e-04	4.00e-04	5.00e-09
13	rs11164144, rs944899		5.00e-09	1.00e-04	1.00e-04
13	rs9508716, rs1275190, rs1275191, rs9508717, rs1314940	*LINC00426*	5.00e-04	1.00e-03	5.00e-09
13	rs3764109, rs9530253	*KLF12*	2.00e-04	5.00e-09	4.00e-04
14	rs11850328, rs174994, rs8014403		8.00e-04	4.00e-04	5.00e-09
16	rs12149339, rs12933074, rs12934725, rs11861289	*RBFOX1*	2.00e-04	5.00e-09	5.00e-09
17	rs1990185, rs17772608, rs11077582, rs12951391, rs978425, rs16977009, rs12941303, rs2158917, rs12941651, rs7213040, rs16977023, rs12709255, rs17767678, rs7214582	AC003051.1	8.00e-04	5.00e-09	1.94e-02
21	rs2831525, rs6516819	LOC101927973	3.00e-04	7.00e-04	5.00e-09

LD, linkage disequilibrium; SNP, single nucleotide polymorphism; IBS, identical by state.

**Table 3.** Group-wise variable selection performance of BBVS and other competing methods

		TPR	TNR	PPV	NPV
Group-level	BBVS	1.000 (0.000)	1.000 (0.000)	0.996 (0.003)	1.000 (0.000)
	gBridge	0.986 (0.005)	1.000 (0.000)	1.000 (0.000)	1.000 (0.000)
	gel	0.980 (0.007)	1.000 (0.000)	1.000 (0.000)	1.000 (0.000)
	grMCP	0.998 (0.002)	1.000 (0.000)	0.972 (0.009)	1.000 (0.000)
	grSCAD	1.000 (0.000)	1.000 (0.000)	0.472 (0.007)	1.000 (0.000)
	grLASSO	1.000 (0.000)	0.990 (0.000)	0.348 (0.007)	1.000 (0.000)
	cMCP	1.000 (0.000)	0.963 (0.002)	0.144 (0.011)	1.000 (0.000)
Element-level	BBVS	0.686 (0.012)	0.999 (0.000)	0.616 (0.009)	1.000 (0.000)
	gBridge	0.643 (0.011)	0.999 (0.000)	0.503 (0.008)	1.000 (0.000)
	gel	0.651 (0.012)	0.999 (0.000)	0.441 (0.009)	1.000 (0.000)
	grMCP	0.306 (0.009)	0.998 (0.000)	0.165 (0.009)	0.999 (0.000)

BBVS, Bayesian bi-level variable selection; TPR, true positive rate; TNR, true negative rate; PPV, positive predictive value; NPV, negative predictive value.

## Supplementary Materials

Supplementary Table 1. p-values of the classic GWAS for the SNPs selected by BBVS.

Supplementary Fig. 1. Trace plot of the regression coefficients θ_1,1 of the first selected SNP-set for 5,000 iterations of the MCMC algorithm. SNP, single nucleotide polymorphism; MCM, Markov chain Monte Carlo.

Supplementary Fig. 2. Trace plot of the regression coefficients θ_1,2 of the first selected SNP-set for 5,000 iterations of the MCMC algorithm. SNP, single nucleotide polymorphism; MCM, Markov chain Monte Carlo.

Supplementary Fig. 3. Trace plot of the regression coefficients θ_1,3 of the first selected SNP-set for 5,000 iterations of the MCMC algorithm. SNP, single nucleotide polymorphism; MCM, Markov chain Monte Carlo.
