# Supplementary material for: Bayesian bi-level variable selection for genome-wide survival study
Source: Genomics Inform. 2023 Jun 28;21(3):e28. doi: 10.5808/gi.23047 (PMC10584651; doi:10.5808/gi.23047)
Supplement: Supplementary Table 1. — p-values of the classic GWAS for the SNPs selected by BBVS. [file gi-23047-Supplementary-Table-1.pdf]

Supplementary Table 1. p-values of the classic GWAS for the SNPs selected by BBVS

Chr	SNP	p-value	Chr	SNP	p-value	Chr	SNP	p-value
2	rs719790	0.3164	6	rs11752339	0.3270	14	rs8021918	0.5641
2	rs12327976	0.8618	6	rs13197064	0.4040	14	rs1959072	0.3407
2	rs1901798	0.2513	6	rs7775551	0.9861	14	rs10162318	0.8161
2	rs10204084	0.0517	6	rs10485285	0.9859	14	rs1889387	0.4666
2	rs4849387	0.2153	7	rs7790719	0.5782	14	rs4981926	0.6330
2	rs10928620	0.9538	7	rs10264235	0.5364	14	rs2378971	0.4542
2	rs7581057	0.4401	7	rs2057921	0.0749	14	rs1278911	0.2441
2	rs1453290	0.4722	7	rs1962784	0.2768	14	rs1112330	0.2732
2	rs1448907	0.2143	7	rs730771	0.0473	14	rs4441184	0.5837
2	rs7593121	0.0687	7	rs17134285	0.1286	14	rs9323156	0.4888
2	rs17044170	0.0252	7	rs7809492	0.7055	14	rs2245010	0.4908
2	rs10190462	0.2316	7	rs41836	0.8298	14	rs712436	0.1065
2	rs1349159	0.1051	8	rs6472375	0.9220	14	rs698305	0.4520
2	rs3748877	0.3267	8	rs7002771	0.8536	14	rs10151509	0.1913
2	rs881639	0.3722	8	rs10113233	0.7785	14	rs10423232	0.5748
2	rs4954516	0.2564	8	rs7842611	0.9949	14	rs4511784	0.2646
2	rs13033419	0.0251	8	rs4384011	0.0221	19	rs6113161	0.7079
2	rs2218707	0.1151	8	rs3134086	0.2034	20	rs12479920	0.8744
2	rs10496773	0.2486	8	rs2055101	0.6470	20	rs6082461	0.3357
4	rs898500	0.2345	8	rs7001811	0.1006	20	rs5756906	0.4817
4	rs1460362	0.2051	8	rs4130891	0.4462	20	rs11704841	0.0350
4	rs7693131	0.9965	8	rs6993813	0.9783	20	rs2213161	0.0347
4	rs2291811	0.0892	8	rs4273881	0.6110	22	rs4821790	0.8672
4	rs2323187	0.0737	8	rs9650075	0.6110	22	rs2330014	0.0871
4	rs7665370	0.2954	8	rs3102735	0.2570	22	rs743517	0.3442
5	rs12657643	0.1743	8	rs1385495	0.2570	22	rs5757171	0.5753
5	rs13158199	0.0757	8	rs11166972	0.1530	22	rs4991801	0.9400
5	rs6863177	0.7617	10	rs10998544	0.0821	22	rs7291684	0.6539
6	rs4360123	0.2606	10	rs2394527	0.5915	22	rs5995615	0.8796
6	rs4496808	0.1849	10	rs1802295	0.8055	22	rs5995628	0.8314
6	rs13213842	0.6269	10	rs953724	0.8721	22	rs2330025	0.6179
6	rs4610573	0.2410	14	rs7156569	0.5278	22	rs7364255	0.2964
6	rs2496690	0.8165	14	rs11850273	0.5908	22	rs1320	0.4895
6	rs9361640	0.6148	14	rs8007488	0.7508	22	rs6001758	0.1721
6	rs9344033	0.8028	14	rs10134871	0.8539			
6	rs2123993	0.9284	14	rs7158021	0.3351			

GWAS, genome-wide association study; SNP, single nucleotide polymorphism; BBVS, Bayesian bi-level

variable selection.
