# Supplementary material for: Bayesian bi-level variable selection for genome-wide survival study
Source: Genomics Inform. 2023 Jun 28;21(3):e28. doi: 10.5808/gi.23047 (PMC10584651; doi:10.5808/gi.23047)
Supplement: Supplementary Fig. 1. — Trace plot of the regression coefficients θ_1,1 of the first selected SNP-set for 5,000 iterations of the MCMC algorithm. SNP, single nucleotide polymorphism; MCM, Markov chain Monte Carlo. [file gi-23047-Supplementary-Fig-1.pdf]

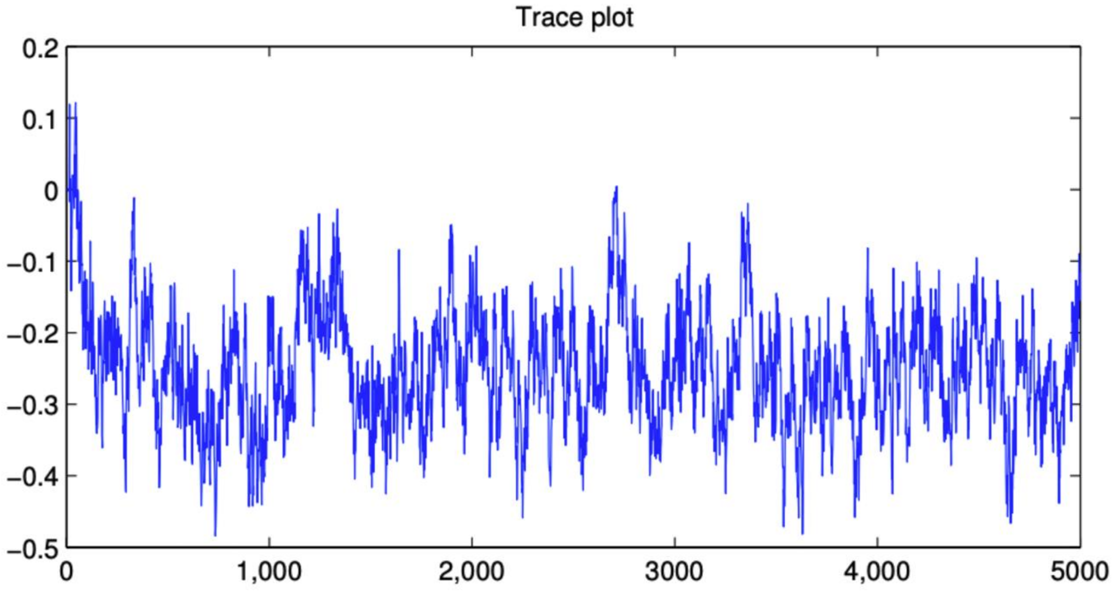

Supplementary Fig. 1. Trace plot of the regression coefficients $\theta_{1,1}$ of the first selected SNP-set for 5,000 iterations of the MCMC algorithm. SNP, single nucleotide polymorphism; MCM, Markov chain Monte Carlo.
